# Standard Heart Rate Variability Parameters—Their Within-Session Stability, Reliability, and Sample Size Required to Detect the Minimal Clinically Important Effect

**DOI:** 10.3390/jcm12093118

**Published:** 2023-04-25

**Authors:** Breda Žunkovič, Nataša Kejžar, Fajko F. Bajrović

**Affiliations:** 1 Clinical Institute of Clinical Neurophysiology, University Medical Centre Ljubljana, Zaloška Cesta 2, 1000 Ljubljana, Slovenia; 2 Faculty of Medicine, University of Ljubljana, Vrazov Trg 2, 1000 Ljubljana, Slovenia; 3 Institute for Biostatistics and Medical Informatics, Faculty of Medicine, University of Ljubljana, Vrazov Trg 2, 1000 Ljubljana, Slovenia; 4 Department of Vascular Neurology and Intensive Neurological Therapy, University Medical Centre Ljubljana, Zaloška Cesta 2, 1000 Ljubljana, Slovenia; 5 Institute of Pathophysiology, Faculty of Medicine, University of Ljubljana, Zaloška Cesta 4, 1000 Ljubljana, Slovenia

**Keywords:** heart rate variability, stability, reliability, within session, sample size, short-term recordings

## Abstract

Many intervention studies assume the stability of heart rate variability (HRV) parameters, and their sample sizes are often small, which can significantly affect their conclusions. The aim of this study is to assess the stability and reliability of standard HRV parameters within a single resting session, and to estimate the sample size required to detect the minimal clinically important effect of an intervention. Heart rate was recorded in 50 adult healthy subjects for 50 min in a seated position. Eight standard HRV parameters were calculated from five evenly spaced 5 min intervals. Stability was assessed by comparing the mean values of HRV parameters between the consecutive five test–retest measurements. Absolute reliability was determined by standard error of measurement, and relative reliability by intraclass correlation coefficient. The sample size required to detect a mean difference of ≥30% of between-subject standard deviation was estimated. As expected, almost all HRV parameters had poor absolute reliability but most HRV parameters had substantial to excellent relative reliability. We found statistically significant differences in almost all HRV parameters between the first 20 min and the last 30 min of the session. The estimated sample size ranged from 19 to 300 subjects for the first 20 min and from 36 to 194 subjects for the last 30 min of the session, depending on the selected HRV parameter. We concluded that optimal HRV measurement protocols in a resting seated position should be performed within the first 20 min or between 20 and 50 min after assuming a resting seated position. Future interventional HRV studies should include a sufficient number of subjects and consider the Bonferroni correction according to the number of selected HRV parameters to achieve an appropriate level of study power and precision.

## 1. Introduction

Heart rate variability (HRV) is a relatively inexpensive and non-invasive method for assessing cardiac autonomic modulation. Reduced HRV is a known independent risk factor for sudden cardiac events and death in the general population [1,2,3], in the elderly [4] and in patients with coronary artery disease [5,6,7,8,9]. It is also a risk stratifier for arrhythmic events and sudden death after myocardial infarction [10,11] and is related to the number of metabolic syndrome components and manifest diabetes [12]. Therefore, the interest in interventional HRV studies in health and disease is growing exponentially. However, the methodology and, thus, the conclusions of many of these studies are controversial.

For a well-designed interventional HRV study, it is important to know the stability and the reliability of HRV parameters, namely the intrinsic physiologic fluctuation over time without any intervention, to be able to determine the significance of the intervention effect on HRV parameters and to estimate the required sample size to detect the minimal clinically important effect of an intervention. Because the effect of certain interventions (e.g., physical, cognitive or pharmacological) is often evaluated in a single session lasting up to one hour, the stability and reliability of HRV parameters within a single resting session (within-session) are of particular importance. The literature search revealed that reports of the stability and reliability of HRV parameters over days/weeks/months far outweigh reports of the stability and reliability of HRV parameters obtained within a single resting session. We found only four studies of the within-session reliability of HRV in short-term recordings in healthy adults [13,14,15,16]. These studies showed substantial relative reliability of HRV parameters [13,14,15,16], and low absolute reliability of HRV parameters [13,15,16]. The time domain parameters of HRV demonstrated greater reliability than the frequency domain parameters of HRV [13,15,16]. In these studies, HRV was analyzed from non-standard 3 and 10 min segments of recordings [13,16], only one or two HRV parameters were evaluated [14,15], and notably, the sample size required to detect the minimal clinically important effect of an intervention on HRV was not estimated.

The aim of this study is to comprehensively evaluate the fluctuation of HRV parameters from standard short-term NN interval recordings and their relative and absolute reliability in healthy, non-athletic young adults during 50 min of rest in a seated position. In addition, based on our results, we calculated the mean detectable change (MDC) and minimal clinically important difference (MCID) [17], and estimated the sample size required for eight standard HRV parameters.

## 2. Materials and Methods

### 2.1. Subjects

Sixty volunteers were recruited for this study. According to a questionnaire completed before the study, three of them smoked, three took hormonal contraceptive pills, four took antihistamines occasionally due to allergies, and one took corticosteroids occasionally for asthma. Apart from these, none of them was taking medication or suffered from any apparent acute or chronic diseases at the time of data acquisition. They exercised between 3 and 6 h per week in their free time.

### 2.2. Study Protocol

Testing was performed between 4 and 7 p.m. in a quiet, illuminated room at a temperature of 22–24 °C with the subjects assuming a resting sitting position. The subjects were asked to abstain from alcohol, caffeine, tobacco, and food for at least 5 h and to refrain from physical exertion for 24 h prior to testing.

Upon arrival at the laboratory, the subjects first completed a questionnaire on age, body mass index, physical activity, and medical history. Then, they were seated in a comfortable chair, and the Polar Wear Link W.I.N.D, attached to a strap, was placed around their chests. NN intervals were measured with three different Polar devices (Polar RS800CX, Polar RS400, and Polar V800, Electro Oy Kempele Finland), which were calibrated before the study to avoid systematic errors. Three Polar devices were used, due to the technical problems. After instrumentation, beat-to-beat NN intervals were recorded continuously for 50 min. Subjects were instructed to breathe and relax naturally, to remain awake, and not to move, talk or perform relaxation techniques. Since there is no agreement on the effect of paced breathing on the reliability of HRV parameters [18], breathing was not paced.

### 2.3. Signal Analysis and Measurements

The 50 min recordings of beat-to-beat NN intervals were imported as ASCII files into a software program (Kubios HRV version 2.0, Department of Physics, University of Kuopio, Finland). The raw NN interval tachograms were visually inspected to assess the quality of signal acquisition. Artifacts were automatically corrected using a piecewise cubic spline interpolation method. The selected level was medium. Low-frequency aperiodic trend components were removed by detrending with the smoothness prior method, with the Lambda set to 500, resulting in a cutoff frequency of 0.035 Hz. Then, the entire 50 min NN interval recording was divided into five 10 min segments (M1, M2, M3, M4, and M5), and the last 5 min of each segment were analyzed in terms of time- and frequency-domain parameters according to the current guidelines for the standards for short-term HRV recordings [19]. The mean NN interval was also analyzed.

As for time-domain HRV parameters, statistical and geometrical parameters were analyzed. The calculated statistical parameters were the standard deviation of NN intervals (SDNN) and the root mean square of successive differences (RMSSD) [19]. Geometrical parameters were derived from the Poincaré plot, where the width and length of the ellipse are denoted as SD1 and SD2, respectively: SD1 is considered to reflect short-term variability, and SD2 is considered to reflect long-term variability [20,21]. Since SD1 and RMSSD are empirically and mathematically identical HRV metrics [22], SD1 was not analyzed.

The frequency domain components of HRV were obtained by a power spectrum analysis of NN intervals using a discrete Fourier transformation. Welch’s window was applied to successive heart rate frequencies to reduce spectral leakage. Then, the area under the low frequency band (LF; 0.04–0.15 Hz) and the area under the high frequency band (HF; 0.15–0.40 Hz) were calculated [19]. The LF and HF components were expressed in absolute values (ms${}^{2}$), and the HF component was also expressed in normalized units (HF*nu* = HF/(HF + LF)). To avoid redundancies, LF power in normalized units and the LF/HF ratio were not analyzed since they are equal to 1 − HF*nu* and (HF*nu*)${}^{-1}$− 1, respectively [23,24].

Since the publication of standards of HRV measurements by Task Force of The European Society of Cardiology and The North American Society of Pacing and Electrophysiology in 1996 [19], new methods have been developed for HRV signal analysis; however, they have provided more information on the complexity and mathematical or physical characteristics of the variability signal than on sympathetic or parasympathetic neural control mechanism and, therefore, have not been vastly integrated into the clinical practice [25,26].

### 2.4. Statistical Analysis

The distribution of HRV parameters in the five measurement segments (M1, M2, M3, M4, and M5) was evaluated using the Shapiro–Wilk test. Values from skewed and/or heteroscedastic measurements were transformed with the natural logarithm (ln). Data were expressed as the mean ± standard deviation and statistical significance was set at α = 0.05. Parameter values that were greater/smaller than the upper/lower quartile ± 1.5 times the interquartile range were further inspected as outliers [27]. Repeated measures ANOVA was performed to analyze the effect of time (independent variables: M1, M2, M3, M4, and M5) on the selected HRV parameter (dependent variable: total HRV, SDNN, RMSSD, HF, LF, SD2, HF*nu*, and mean NN). If the sphericity assumption was not met, Greenhouse–Geisser sphericity correction was used. Post hoc comparisons using Benjamini–Hochberg correction were performed for HRV parameters that significantly differ between measurements.

Relative reliability was assessed with the intraclass correlation coefficient (ICC), calculated according to Shrout and Fleiss [28]. As suggested by Atkinson and Nevill [29], ICC > 0.8 was considered to have good to excellent reliability and ICC 0.6-0.8 was considered to have substantial reliability. Absolute reliability was assessed using the standard error of measurement (SEM), calculated according to Wier [30]. Next, the 95% limits of random variation and the corresponding minimal detectable changes (MDC) were derived. For log transformed variables, these limits were back transformed (exponentiated). For a better comparison between studies, the coefficient of variation (CV) was also calculated.

According to Pinna et al. [31], a change of ≥30% of between-subject SD was considered a relevant change or minimal clinically important difference (MCID). In the computation of the sample size, we assumed a two-tailed one sample t-test. Sample size estimates were calculated for each HRV parameter separately (α=0.05 with a power of 80%) and for all eight HRV parameters together (in that case, the significance level was corrected for multiple parameters—Bonferroni, α=0.05/8).

For further explanation of the statistical analysis, please see Appendix A.

IBM SPSS Statistics 18.0 was used for the descriptive statistics and calculation of the reliability, whereas PS (Power and Sample Size Calculation version 3.1.2) was used for sample size estimation [32].

## 3. Results

Ten subjects were excluded from the analysis because visual inspection of the NN tachograms revealed poor signal acquisition, reducing the final study population to fifty subjects (31 males, age 23 (±4), BMI 23.2 kg/m${}^{2}$ (±2.8)).

**Figure A1:**
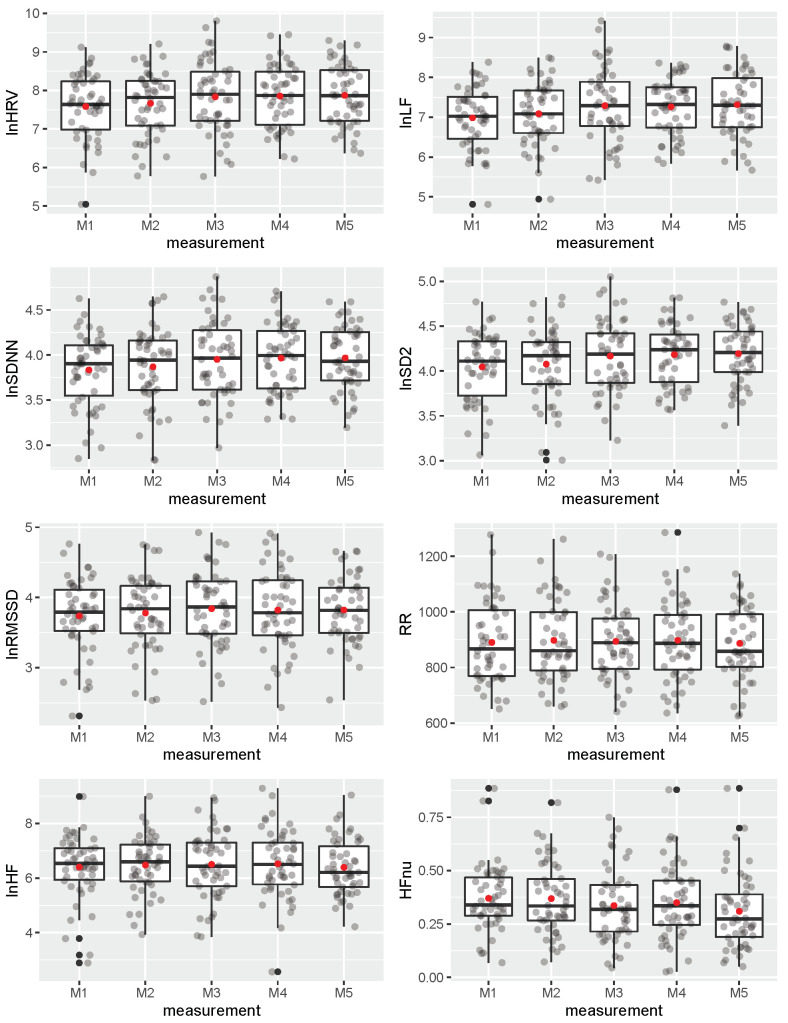
Each boxplot presents the measurement data of all 50 subjects for a single HRV parameter (log-transformed values for SDNN, RMSSD, total HRV, LF, HF and SD2, and original values for HF*nu* and mean NN interval). The red dots are the mean values, the grey dots present the individuals’ single measurement value. M1 = first 10 min, M2 = second 10 min, M3 = third 10 min, M4 = fourth 10 min, M5 = fifth 10 min of the 50 min NN interval recording.

The means and standard deviations of HRV parameters in five 5 min segments (M1–M5) are presented in Table 1 (for individual data, see Figure A1). All HRV parameters, except the mean NN interval and HF*nu*, exhibited a right-skewed distribution and/or heteroscedasticity. For those, logarithmically transformed values were analyzed.

One of the subjects appeared as an outlier on the HF*nu* parameter in all segments except M3, and was therefore excluded from further statistical analysis of this parameter.

### 3.1. Stability of HRV

ANOVA (see Table A1) showed a non-significant main effect of time on RMSSD (F = 2.205, *p* = 0.088), HF (F = 0.841, *p* = 0.501), and mean NN (F = 0.506, *p* = 0.666). For all other HRV parameters, the main effect of time was significant, and post hoc analyses of pairwise comparisons are shown in Table A2.

Exploratory analysis of the comparison of means showed the following patterns: the mean values of ln total HRV, ln SDNN, ln LF, and ln SD2 were statistically significantly higher in segments M3, M4, and M5 than in segments M1 and M2; and the mean value of HF*nu* was statistically significantly lower in segment M5 than in segments M1 and M2 (Table 1 and Table A2). Therefore, the reliability indices and sample sizes for the two sets of segments were assessed separately (M1&M2 and M3&M4&M5).

### 3.2. Reliability Indices

Reliability indices are shown in Table 2 for segments M1 and M2, and in Table 3 for segments M3, M4, and M5. All reliability indices were better in segments M1 and M2 than in segments M3, M4, and M5, except SEM for the mean NN parameter and ICC for the HF*nu* parameter.

The 95% interval of random variation and MDC derived from SEM showed large differences between segments of measurements (M*i*/M*j*) for all HRV parameters. For heteroscedastic parameters, ln and exponentiated values for MDC_(95%)_ are shown (Table 2 and Table 3). In the worst case of absolute reliability (ln LF parameter in segments M3, M4, and M5), the limits of the ratio of a repeated parameter (M*i*/M*j*) were 0.24–4.06, indicating that this parameter can decrease by up to 76% or increase by up to 306% in the repeated measurement compared with the first measurement. The extent of change in an HRV parameter due to an experimental intervention must therefore exceed these limits to achieve 95% confidence in its authenticity.

The ICC indices showed substantial–excellent relative reliability (ICC 0.6–1) for most of the HRV parameters (Table 2 and Table 3). There was a trend toward better absolute and relative reliability for time domain parameters than for frequency domain parameters.

### 3.3. Sample Size

Sample size estimates per HRV parameter reflected the variations in reliability characteristics for the first 20 min and the last 30 min of the 50 min NN interval recording and are shown in Table 4.

Allowing for ≥30% of between-subject SD as MCID for all analyzed parameters, and applying the Bonferroni correction, sample sizes of at least 300 subjects for the first 20 min and 194 subjects for the last 30 min of the 50 min NN interval recording would be required if all eight parameters of the HRV are to be analyzed (α = 0.05/8, β = 0.2). However, if only one HRV parameter is to be analyzed (and thus without applying the Bonferroni correction), then—for the parameter total HRV—46 subjects would be required for the first 20 min, and 84 subjects would be required for the last 30 min of the recording (α = 0.05, β = 0.2). In this case, the analysis must be limited to a single HRV parameter.

As shown in Table 4, the required sample size for the first 20 min is doubled if the parameter HF*nu* is included in the statistical analysis because of its poor reliability indices. However, if the HF*nu* is excluded from the analysis (not shown in Table 4), a sample size of at least 137 subjects for the first 20 min, and 183 subjects for the last 30 min of the 50 min NN interval recording would be required (α = 0.05/7, β = 0.2), reflecting the better reliability of most HRV parameters within the first 20 min of the 50 min NN interval recording.

## 4. Discussion

This study showed marked fluctuation of the HRV parameters during 50 min recording in a sitting position, with a significant change after 20 min. As expected, HRV parameters showed low absolute reliability and substantial to excellent relative reliability. The main motivation for this study was to estimate the sample sizes required to detect minimal clinical important change in selected HRV parameters, which proved to be considerably high compared with the sample size chosen in many interventional HRV studies.

### 4.1. The Magnitude of HRV Parameters

In the present study, the mean values of HRV parameters in the time and frequency domains were within the range of a previous study on reliability in a similar population of subjects [33]. However, the mean values of frequency domain HRV parameters in the present study were higher than in previous studies reporting normal HRV values, probably due to older population of subjects in previous studies [34,35,36,37].

### 4.2. Stability of HRV Parameters

The means of SDNN, total HRV, LF and SD2—HRV parameters reflecting sympathetic–parasympathetic heart rate modulation [19,38]—were significantly higher during the last 30 min (measurements M3, M4, and M5) than during the first 20 min of NN interval recording (measurements M1 and M2).

Similarly, a previous study reported a significant increase in HRV parameters influenced by sympathetic-parasympathetic modulation (SDNN, SD2, total HRV, LF, LF*nu*, LF/HF) within the last 20 min of a 40 min RR interval recording in supine, sitting, and standing positions (each position on a different day) [13]. As suggested by the authors [13], the lack of a significant change (RMSSD) or even a decrease (HF) in parameters reflecting parasympathetic modulation of heart rate [19,21,38,39] may indicate a steady increase in sympathetic modulation during the 40 min of rest in a seated position. This is supported also by our results, namely, that parameters indicating parasympathetic modulation (RMSSD, HF, and HF*nu*) did not significantly increase during 50 min recording; on the contrary, HF*nu* significantly decreased in the last measurement. A recent study also showed that the RMSSD parameter reached a plateau within the first 40 min of a 50 min recording in the supine position, and decreased thereafter [15].

The results of the present and other studies [13,15] suggest, that sympathetic heart rate modulation continuously increases during resting position and doing nothing, possibly due to fatigue and/or restlessness. Consequently, the first part of NN interval recording may be significantly different from the second part, merely due to physiological fluctuation of HRV parameters, which may be important in developing an optimal measurement protocol.

### 4.3. Reliability Indices

#### 4.3.1. Absolute Reliability

We found large random variations in all HRV parameters between adjacent segments of recordings from the same subject (SEM and MDC_(95%)_), which implies that HRV is not optimal to detect intervention effects in individual subjects. This is consistent with previous studies of the within-session reliability of HRV [13,15], which showed low absolute reliability of HRV parameters.

The main clinical implication of large 95% random variation limits is that a substantial change is required for at least 95% certainty that an intervention has a real effect [40]. Therefore, HRV measurements from short-term recordings may be of limited value for evaluating the efficacy of interventions in individual subjects, although the more reliable time domain parameters may be more appropriate for this purpose than their frequency domain counterparts [41,42].

#### 4.3.2. Relative Reliability

In the first 20 min, ICC values were 0.60–0.93 for all HRV parameters, except HF*nu*, indicating that relative reliability was at least substantial [29,43]. Similar ICC values were found in two within-session reliability studies on healthy young adults in supine, sitting, and standing rest positions [13,15]. One within-session reliability study reported higher values for ICCs, but the protocol included orthostatic stimulation [44]. Another within-session reliability study reported higher ICC values for LF and HF parameters, but the analysis was performed on 60 consecutive 5 min segments within 15 min RR interval recordings (5 min window shifted by 10 s), meaning that 2 consecutive 5 min segments were almost identical [14].

According to previous reports [13,14,15], the present study showed generally higher ICC values of HRV parameters reflecting parasympathetic heart rate modulation (0.83–0.93 for RMSSD and HF compared with 0.60–0.89 for SDNN, total HRV, LF, and SD2), making them suitable for test–retest study protocols on a group of subjects since the estimated sample size could be smaller. The lowest ICC value for HF*nu* was due to a relatively high random error, most likely because this parameter “contains” the error from both LF and HF, which was also observed in a similar previous study [31].

### 4.4. Sample Size Estimation

As already acknowledged by Pinna et al., an important implication of reliability studies is the estimation of the sample size required to detect a significant change in the measurement average with a certain level of significance and power in a test–retest experiment [31]. The main challenge is to define clinically significant change in mean values (namely MCID) for HRV parameters, as there are no epidemiological anchor-based studies in this regard yet. In the absence of criteria for a “clinically relevant change”, Pinna et al. [31] arbitrarily adopted a criterion of change of ≥30% of between-subject SD. On the other hand, Tannus et al. defined the minimal statistically significant difference by using within-subject SD, which, in our opinion, is not appropriate for estimating the difference in population averages (e.g., using *t*-tests).

To our knowledge, this is the first study to estimate the sample size for the purpose of within-session (interventional) HRV studies, and also the first to consider the Bonferroni correction. Table 4 shows that the required sample size almost halved when the Bonferroni correction was not applied, but in this case, the researcher should limit the analysis to a single HRV parameter. If a researcher decides to study only one HRV parameter, then the mean NN or one of the time domain parameters would require the smallest sample size. In addition to the selection of HRV parameter(s), the sample size estimate depends on the population studied (the more homogeneous the population, the lower the ICC, and the larger the sample size), the time of recording, and the minimum difference in averages that one attempts to detect. The calculated required sample size in this study was larger than most interventional HRV studies have considered.

The estimated sample sizes in the present study were larger than in previous inter-day reliability studies [31,44,45,46], in which no correction of α for multiple parameters was applied. In addition, previous studies included orthostatic stimulation [44], pipetting [45], or patients with diabetes mellitus [46], which may influence the reliability of the HRV parameters and, therefore, the estimated sample size.

HRV parameters generally decrease with age, but RMSSD and pNN50 decrease from age 40 to 60 years and then increase after age 70 years [47]. HF power is lower and LF power is higher in males than females [48]. HRV parameters are lower in the standing than in the supine position [49]. HRV parameters decrease with decreasing health status [50], but they may be increased in some cases, such as in post-COVID-19 [51]. The magnitude of HRV parameters can affect CV and ICC values [42]. This can have a particular impact when reliability indices are used to estimate sample sizes for studies. A power calculation based on the reliability indices derived from healthy subjects may result in a significant underestimation of the required sample size and, thus, a lower statistical power.

### 4.5. Limitations

Our study has several limitations. First, measurements were performed in the afternoon between 4 and 7 p.m. throughout the year. HRV parameters have been shown to follow a circadian rhythm similar to other physiological parameters [52,53,54,55], with the lowest values recorded in the afternoon [56]. HRV parameters have also been shown to vary with season [57,58]. Overall, this could mean that the variance of our results could be higher (and relative reliability lower and the required sample size higher) than in the case where all measurements are taken at the same time of day within a month. Second, the present study included a uniform group of young healthy subjects, which could lead to a lower relative reliability and thus a larger estimated sample size required to detect differences in means between two independent groups. Therefore, our results on within-session reliability of HRV parameters and estimated sample size cannot be generalized to other populations. Third, the use of three different Polar devices may have caused a systematic error. To reduce this risk, we calibrated the devices before conducting the experiment. Fourth, breathing was not paced in our study, which may have also influenced the results. However, there is no agreement in the literature on the effect of paced breathing on the reliability of HRV parameters [18]. Some studies reported an improvement in relative reliability of the HF band [59] and LF*nu* parameter [31,59], a decrease in relative reliability of the LF band [59], and an improvement in the absolute reliability of both the HF and LF bands [60] after paced breathing, while others found no significant influence on the reliability of HRV parameters [18,37,61,62,63].

## 5. Conclusions

We found significant differences in the mean values of most HRV parameters between measurements during the first 20 min compared with measurements during the last 30 min of recording. Therefore, optimal measurement protocols in a resting sitting position should be performed within the first 20 min or between 20 and 50 min after assuming a resting sitting position, and measurement protocols that include the first 20 min and the last 30 min of the 50 min in a resting position should account for the spontaneous increase in most HRV parameters.

The low absolute reliability of all HRV parameters, particularly in the frequency domain, and good relative reliability of almost all HRV parameters are consistent with earlier studies [13,14,15,16]; this suggests that this method may not be suitable for the assessment of treatment or intervention effects in individual subjects but rather for test–retest study protocols on a group of subjects. Since ICC values of the HRV parameters reflecting parasympathetic modulation of the heart rate (RMSSD and HF) were higher than those of other HRV parameters, it can be suggested that these HRV parameters are most suitable for test–retest experiments. On the other hand, the HF*nu* parameter is the least appropriate for test–retest experiments because its ICC values were the lowest.

The sample size estimation in this study is the pilot one that considers the within-session stability of HRV parameters and the Bonferroni correction. It provides accurate numbers that can guide future interventional HRV studies and enable researchers to assess the power of previous interventional within-session studies. Considering all the findings of this study, it is obvious that the resting protocol urgently needs to be standardized if we are to design a valid study protocol and compare the results of different HRV studies.

## Figures and Tables

**Table 1:** Descriptive characteristics of original values and log-transformed (ln) values of HRV parameters [mean (±SD)].

	M1	M2	M3	M4	M5
ln total HRV	7.58 (±0.84)	7.66 (±0.81)	7.83 (±0.93)	7.85 (±0.79)	7.87 (±0.77)
ln SDNN	3.83 (±0.40)	3.87 (±0.43)	3.95 (±0.43)	3.97 (±0.37)	3.97 (±0.35)
ln RMSSD	3.74 (±0.53)	3.78 (±0.53)	3.84 (±0.54)	3.82 (±0.57)	3.82 (±0.47)
ln HF	6.4 (±1.16)	6.48 (±1.03)	6.5 (±1.23)	6.52 (±1.25)	6.39 (±1.0)
ln LF	6.98 (±0.75)	7.08 (±0.77)	7.29 (±0.93)	7.26 (±0.7)	7.31 (±0.8)
ln SD2	4.04 (±0.38)	4.08 (±0.4)	4.17 (±0.4)	4.18 (±0.34)	4.19 (±0.33)
HF*nu*	0.37 (±0.15)	0.37 (±0.16)	0.34 (±0.17)	0.35 (±0.17)	0.31 (±0.18)
mean NN(ms)	890 (±144)	897 (±143)	894 (±128)	898 (±139)	887 (±129)

M1 the last 5 min of the first 10 min block, M2 the last 5 min of the second 10 min block, M3 the last 5 min of the third 10 min block, M4 the last 5 min of the fourth 10 min block, M5 the last 5 min of the fifth 10 min block of the 50 min NN interval recording.

**Table 2:** Reliability indices for HRV parameters in the first 20 min of NN interval recording (M1 and M2 measurements).

	SEM	CV%	MDC_(95%)_	MDC_(95%)_ anti-log	ICC (95% CI)
ln total HRV	0.37 ln ms${}^{2}$	4.9	±1.03 *	0.36–2.77	0.80 (0.67–0.88)
ln SDNN	0.14 ln ms	3.6	±0.39 *	0.69–1.46	0.89 (0.81–0.93)
ln RMSSD	0.14 ln ms	3.7	±0.39 *	0.67–1.49	0.93 (0.87–0.96)
ln HF	0.44 ln ms${}^{2}$	6.8	±1.22 *	0.30–3.36	0.84 (0.74–0.91)
ln LF	0.44 ln ms${}^{2}$	6.3	±1.22 *	0.30–3.36	0.67 (0.49–0.80)
ln SD2	0.15 ln ms	3.7	±0.42 *	0.66–1.51	0.86 (0.76–0.92)
HF*nu*	0.10	27.7	±0.28		0.49 (0.25–0.68)
Mean NN	32 ms	3.6	±89 ms		0.95 (0.91–0.97)

SEM standard error of measurement, CV coefficient of variance, MDC_(95%)_ minimal detectable change with a 95% confidence interval, ICC (95% CI) intraclass correlation coefficient with a 95% confidence interval. * Heteroscedastic HRV parameter; therefore, ln(MDC).

**Table 3:** Reliability indices for HRV parameters in the last 30 min of NN interval recording (M3, M4, and M5 measurements).

	SEM	CV%	MDC_(95%)_	MDC_(95%)_ anti-log	ICC (95% CI)
ln total HRV	0.48 ln ms${}^{2}$	6.1	±1.33 *	0.26–3.78	0.67 (0.54–0.78)
ln SDNN	0.14 ln ms	3.5	±0.39 *	0.67–1.49	0.86 (0.78–0.91)
ln RMSSD	0.20 ln ms	5.2	±0.55 *	0.57–1.75	0.85 (0.78–0.91)
ln HF	0.48 ln ms${}^{2}$	7.4	±1.33 *	0.26–3.78	0.83 (0.75–0.90)
ln LF	0.51 ln ms${}^{2}$	7.0	±1.41 *	0.24–4.06	0.60 (0.44–0.73)
ln SD2	0.15 ln ms	3.6	±0.42 *	0.66–1.52	0.82 (0.74–0.89)
HF*nu*	0.10	30.1	±0.28		0.63 (0.48–0.75)
Mean NN	42 ms	4.7	±116 ms		0.90 (0.84–0.94)

SEM standard error of measurement, CV coefficient of variance, MDC_(95%)_ minimal detectable change with a 95% confidence interval, ICC (95% CI) intraclass correlation coefficient with a 95% confidence interval. * Heteroscedastic HRV parameter, therefore ln(MDC).

**Table 4:** Estimated sample sizes for HRV parameters in the first 20 min and in the last 30 min of the 50 min NN interval recording, with and without applying the Bonferroni correction (B.C.). The estimated sample size considerably decreases if only a single HRV parameter is considered (i.e., B.C. is not applied).

	First 20 min			Last 30 min		
	MCID_(30%)_	Sample Size	Sample Size	MCID_(30%)_	Sample Size	Sample Size
		(B.C. Applied)			(B.C. Applied)	
ln total HRV	0.22 ln ms${}^{2}$	75	46	0.21 ln ms${}^{2}$	144	84
ln SDNN	0.12 ln ms	39	23	0.10 ln ms	51	33
ln RMSSD	0.15 ln ms	26	16	0.14 ln ms	54	34
ln HF	0.30 ln ms${}^{2}$	58	36	0.32 ln ms${}^{2}$	62	37
ln LF	0.19 ln ms${}^{2}$	144	86	0.19 ln ms${}^{2}$	194	115
ln SD2	0.11 ln ms	51	31	0.10 ln ms	67	37
HF*nu*	0.03	300	176	0.04	171	100
Mean NN	42 ms	19	11	37 ms	36	22

## Data Availability

The data presented in this study are available on reasonable request from the corresponding author.

## Appendix A. Statistical Method

Relative reliability was assessed using the intraclass correlation coefficient (ICC), calculated according to Shrout and Fleiss [28] as follows:

$$\mathrm{ICC}=\frac{\mathrm{BMS}-\mathrm{WMS}}{\mathrm{BMS}+(k-1)\mathrm{WMS}}$$

where BMS is between-subject mean squares, WMS is within-subject mean squares, given by one-way ANOVA, and *k* is the number of subsequent measurements (in our case, 5 or less). A 95% confidence interval around the ICC was calculated. As suggested by Atkinson and Nevill [29], ICC > 0.8 was considered to be of good to excellent reliability, and ICC 0.6–0.8 was considered to be of substantial reliability.

Absolute reliability was assessed using the standard error of measurement (SEM), calculated according to Wier [30] as follows:

$$\mathrm{SEM}={\mathrm{SD}}_{\mathrm{total}}\times \sqrt{1-\mathrm{ICC}}$$

where SD_total_ applies to the entire sample given by $\sqrt{\frac{{\mathrm{SS}}_{\mathrm{total}}}{k\times n-1}}$ (SS_total_ is the total sum of squares for ANOVA, *k* is the number of subsequent measurements, and *n* is the number of subjects). The SEM value was additionally double checked with the $\sqrt{\mathrm{WMS}}$ given by one-way ANOVA to minimize the effect of between-subject variability on the estimate of SEM. Next, the 95% limits of random variation and the corresponding minimal detectable changes (MDC) were derived from the product of $\mathrm{SEM}\times 1.96\times \sqrt{2}$. For log-transformed variables, these limits were back transformed (exponentiated).

For a better comparison between studies, the coefficient of variation (CV) was also calculated as follows:

$$\mathrm{CV}=\frac{\mathrm{SEM}}{\mathrm{mean}}$$

where mean is the mean value of a given HRV parameter.

For sample size estimation, the minimal clinically important difference (MCID) in a test–retest experiment was considered to be a change of ≥30% of between-subject SD (according to Pinna et al. [31]), and the between-subject SD was calculated as follows:

$$S_{B}=\sqrt{\frac{\mathrm{ICC}}{1-\mathrm{ICC}}\times {S_{W}}^{2}}$$

where S_B_ is between-subject SD, and S_W_ is within-subject SD, which is actually SEM.

## Appendix B. Results

**Table A1:** ANOVA of mean values of all HRV parameters. Additional pairwise comparisons were performed only for HRV parameters with significant results of ANOVA. * *p* < 0.05.

	F	*p*
ln total HRV	4.36	0.004 *
ln SDNN	8.106	0.011 *
ln RMSSD	2.205	0.088
ln HF	0.841	0.501
ln LF	4.702	0.001 *
ln SD2	9.318	0.006 *
HF*nu*	3.399	0.01 *
mean NN	0.506	0.666

* Statistically significant

**Table A2:** Stability of HRV parameters across 50 min recording. *p* values of ANOVA’s post hoc analyses for pairwise comparisons of means for all possible pairs of measurements for those HRV parameters, in which the main effect of time was significant. Benjamini–Hochberg method was used in calculation of *p* values. * *p* < 0.05.

	M1M2	M1M3	M1M4	M1M5	M2M3	M2M4	M2M5	M3M4	M3M5	M4M5
ln total HRV	0.379	0.027 *	0.007 *	0.027 *	0.047 *	0.032 *	0.051	0.874	0.874	0.874
ln SDNN	0.316	0.003 *	<0.001 *	0.001 *	0.013 *	0.004 *	0.013 *	0.660	0.660	0.953
ln LF	0.354	0.014 *	0.012 *	0.008 *	0.045 *	0.076	0.031 *	0.817	0.817	0.757
ln SD2	0.427	0.003 *	<0.001 *	<0.001 *	0.005 *	0.003 *	0.003 *	0.680	0.470	0.688
HF*nu*	0.952	0.162	0.416	0.019 *	0.184	0.438	0.019 *	0.504	0.343	0.112

M1 the last 5 min of the first 10 min block, M2 the last 5 min of the second 10 min block, M3 the last 5 min of the third 10 min block, M4 the last 5 min of the fourth 10 min block, M5 the last 5 min of the fifth 10 min block of the 50 min NN interval recording.

## References

[1] Tsuji H., Larson M., Venditti F., Manders E., Evans J.C., Feldman C., Levy D. (1996). Impact of reduced heart rate variability on risk of cardiac events. The Framingham study. Circulation.

[2] Lee C.H., Lee J.H., Son J.W., Kim U., Park J.S., Lee J., Shin D.G. (2018). Normative values of short-term heart rate variability parameters in Koreans and their clinical value for the prediction of mortality. Hear. Lung Circ..

[3] Maheshwari A., Norby F.L., Soliman E.Z., Adabag S., Whitsel E.A., Alonso A., Chen L.Y. (2016). Low heart rate variability in a 2 min electrocardiogram recording is associated with an increased risk of sudden cardiac death in the general population: The atherosclerosis risk in communities study. PLoS ONE.

[4] Tsuji H., Venditti F., Manders E., Evans J.C., Larson M., Feldman C., Levy D. (1994). Reduced heart rate variability and mortality risk in an elderly cohort. The Framingham study. Circulation.

[5] van Boven A., Jukema J., Haaksma J., Zwinderman A., Crijns H., Lie K. (1998). Depressed heart rate variability is associated with events in patients with stable coronary artery disease and preserved left ventricular function. Am. Heart J..

[6] Nolan J., Batin P., Andrews R., Lindsay S., Brooksby P., Mullen M., Baig W., Flapan A., Cowley A., Prescott R.J. (1998). Prospective study of heart rate variability and mortality in chronic heart failure: Results of the United Kingdom heart failure evaluation and assessment of risk trial. Circulation.

[7] Fang S.C., Wu Y.L., Tsai P.S. (2020). Heart rate variability and risk of all-cause death and cardiovascular events in patients with cardiovascular disease: A meta-analysis of cohort studies. Biol. Res. Nurs..

[8] Sessa F., Anna V., Messina G., Cibelli G., Monda V., Marsala G., Ruberto M., Biondi A., Cascio O., Bertozzi G. (2018). Heart rate variability as predictive factor for sudden cardiac death. Aging.

[9] Kleiger R.E., Miller J.P., Bigger J.T., Moss A.J. (1987). Decreased heart rate variability and its association with increased mortality after acute myocardial infarction. Am. J. Cardiol..

[10] Hohnloser S.H., Klingenheben T., Zabel M., Li Y.G. (1997). Heart rate variability used as an arrhythmia risk stratifier after myocardial infarction. Pacing Clin. Electrophysiol..

[11] Reed M.J., Robertson C., Addison P. (2005). Heart rate variability measurements and the prediction of ventricular arrhythmias. QJM.

[12] Azulay N., Olsen R.B., Nielsen C.S., Stubhaug A., Jenssen T.G., Schirmer H., Frigessi A., Rosseland L.A., Tronstad C. (2022). Reduced heart rate variability is related to the number of metabolic syndrome components and manifest diabetes in the sixth Tromsø study 2007–2008. Sci. Rep..

[13] Young F., Leicht A. (2011). Short-term stability of resting heart rate variability: Influence of position and gender. Appl. Physiol. Nutr. Metab..

[14] Yildiz M., Doma S. (2018). Effect of spontaneous saliva swallowing on short-term heart rate variability (HRV) and reliability of HRV analysis. Clin. Physiol. Funct. Imaging.

[15] Cipryan L. (2016). Within-session stability of short-term heart rate variability measurement. J. Hum. Kinet..

[16] Gisselman A.S., D’Amico M., Smoliga J.M. (2020). Optimizing inter-session reliability of heart rate variability-the effects of artefact correction and breathing type. J. Strength Cond. Res..

[17] de Vet H., Terwee C. (2009). The minimal detectable change should not replace the minimal important difference. Letters to the editor. J. Clin. Epidemiol..

[18] Kobayashi H. (2009). Does paced breathing improve the reproducibility of heart rate variability measurements?. J. Physiol. Anthropol..

[19] Malik M., Task Force of the European Society of Cardiology the North American Society of Pacing Electrophysiology (1996). Heart rate variability: Standards of measurement, physiological interpretation, and clinical use. Circulation.

[20] Tarvainen M.P., Niskanen J.-P., Lipponen J.A., Ranta-aho P.O., Karjalainen P.A. (2014). Kubios HRV–Heart rate variability analysis software. Comput. Methods Programs Biomed..

[21] Kamen P., Krum H., Tonkin A. (1996). Poincaré plot of heart rate variability allows quantitative display of parasympathetic nervous activity in humans. Clin. Sci..

[22] Ciccone A.B., Siedlik J.A., Wecht J.M., Deckert J.A., Nguyen N.D., Weir J.P. (2017). Reminder: RMSSD and SD1 are identical heart rate variability metrics. Muscle Nerve.

[23] Burr R. (2007). Interpretation of normalized spectral heart rate variability indices in sleep research: A critical review. Sleep.

[24] Heathers A. (2014). Everything Hertz: Methodological issues in short-term frequency-domain HRV. Front. Physiol..

[25] Shaffer F., Ginsberg J.P. (2017). An overview of heart rate variability metrics and norms. Front. Public Health.

[26] Sassi R., Cerutti S., Lombardi F., Malik M., Huikuri H.V., Peng C.K., Schmidt G., Yamamoto Y., Reviewers: D., Gorenek B. (2015). Advances in heart rate variability signal analysis: Joint position statement by the e-Cardiology ESC Working Group and the European Heart Rhythm Association co-endorsed by the Asia Pacific Heart Rhythm Society. Europace.

[27] Wilcox R. (2010). Fundamentals of Modern Statistical Methods Substantialy Improving Power and Accuracy.

[28] Shrout P., Fleiss J. (1979). Intraclass correlations: Uses in assessing rater reliability. Psychol. Bull..

[29] Atkinson G., Nevill A. (1998). Statistical methods for assessing measurement error (reliability) in variables relevant to sports medicine. Sport. Med..

[30] Wier J. (2005). Quantifying test-retest reliability using the intraclass correlation coefficient and SEM. J. Strength. Cond. Res..

[31] Pinna G., Maestri R., Torunski A., Danilowicz-Szymanowicz L., Szwoch M., la Rovere M., Raszak G. (2007). Heart rate variability measures: A fresh look at reliability. Clin. Sci..

[32] Dupont W., Plummer W. (2014). Power and Sample Size Calculations.

[33] Tannus L., Sperandei S., Montenegro R., Carvalho V., Pedrosa H., Felix M., Canani L., Zucatti A., de Oliveira D., Rea R. (2013). Reproducibility of methods used for the assessment of autonomous nervous system’s function. Auton. Neurosci..

[34] Voss A., Heitman A., Schroeder R., Peters A., Perz S. (2012). Short-term heart rate variability—age dependence in healthy subjects. Physiol. Meas..

[35] Hemingway H., Shipley M., Brunner E., Britton A., Malik M., Marmot M. (2005). Does autonomic function link social position to coronary risk? The Whitehall II study. Circulation.

[36] Kuo T.B., Lin T., Yang C.C., Li C.L., Chen C.F., Chou P. (1999). Effect of aging on gender differences in neural control of heart rate. Am. J.-Physiol.-Heart Circ. Physiol..

[37] Sinnreich R., Kark J., Friedlander Y., Sapoznikov D., Luria M. (1998). Five minute recordings of heart rate variability for population studies: Repeatability and age–sex characteristics. Heart.

[38] Akselrod S., Gordon D., Madwed J., Snidman N., Shannon D., Cohen R. (1985). Hemodynamic regulation: Investigation by spectral analysis. Am. J. Physiol..

[39] Martinmaki K., Rusko H., Kooistra L., Kettunen J., Saalasti S. (2006). Intraindividual validation of heart rate variability indexes to measure vagal effects on hearts. Am. J. Physiol. Heart Circ. Physiol..

[40] Flouris A., Poirier M., Bravi A., Wright-Beatty H., Herry C., Seely A., Kenny G. (2014). Changes in heart rate variability during the induction and decay of heat acclimation. Eur. J. Appl. Physiol..

[41] Dantas E., Goncalves C., Silva A., Rodrigues S., Ramos M., Andreao R., Pimentel E., Lunz W., Mill J. (2010). Reproducibility of heart rate variability parameters measured in healthy subjects at rest and after a postural change maneuver. Braz. J. Med. Biol. Res..

[42] Sandercock G., Bromley P., Brodie D. (2005). The reliability of short-term measurements of heart rate variability. Int. J. Cardiol..

[43] Donner A., Eliasziw M. (1987). Sample size requirements for reliability studies. Stat. Med..

[44] Cipryan L., Litschmannova M. (2013). Intra-day and inter-day reliability of heart rate variability measurement. J. Sport Sci..

[45] Hallman D.M., Srinivasan D., Mathiassen S.E. (2015). Short-and long-term reliability of heart rate variability indices during repetitive low-force work. Eur. J. Appl. Physiol..

[46] Sacre J., Jellis C., Marwick T., Coombes J. (2012). Reliability of heart rate variability in patients with type 2 diabetes mellitus. Diabet. Med..

[47] Almeida-Santos M.A., Barreto-Filho J.A., Oliveira J.L.M., Reis F.P., da Cunha Oliveira C.C., Sousa A.C.S. (2016). Aging, heart rate variability and patterns of autonomic regulation of the heart. Arch. Gerontol. Geriatr..

[48] Koenig J., Thayer J.F. (2016). Sex differences in healthy human heart rate variability: A meta-analysis. Neurosci. Biobehav. Rev..

[49] da Cruz C.J.G., Porto L.G.G., da Silva Rolim P., de Souza Pires D., Garcia G.L., Molina G.E. (2019). Impact of heart rate on reproducibility of heart rate variability analysis in the supine and standing positions in healthy men. Clinics.

[50] Jarczok M.N., Weimer K., Braun C., Williams D.P., Thayer J.F., Guendel H.O., Balint E.M. (2022). Heart rate variability in the prediction of mortality: A systematic review and meta-analysis of healthy and patient populations. Neurosci. Biobehav. Rev..

[51] Asarcikli L.D., Hayiroglu M.İ., Osken A., Keskin K., Kolak Z., Aksu T. (2022). Heart rate variability and cardiac autonomic functions in post-COVID period. J. Interv. Card. Electrophysiol..

[52] Boudreau P., Yeh W., Dumont G., Boivin D. (2012). A circadian rhythm in heart rate variability contributes to the increased cardiac sympathovagal response to awakening in the morning. Chronobiol. Int..

[53] Boudreau P., Dumont G., Kin N., Walker C., Boivin D. Correlation of heart rate variability and circadian markers in humans. Proceedings of the 2011 Annual International Conference of the IEEE Engineering in Medicine and Biology Society.

[54] Burgess J., Trinder J., Kim Y., Luke D. (1997). Sleep and circadian influences on cardiac autonomic nervous system activity. Am. J. Physiol..

[55] Scheer F., Hu K., Evoniuk H., Kelly E., Malhotra A., Hilton M., Shea S. (2010). Impact of the human circadian system, exercise, and their interaction on cardiovascular function. Proc. Natl. Acad. Sci. USA.

[56] Rodrigues-Colon S., He F., Bixler E., Fernandez-Mendoza J., Vgontzas A., Berg A., Kawasawa Y., Liao D. (2014). The circadian pattern of cardiac autonomic modulation and obesity in adolescents. Clin. Auton. Res..

[57] Kristal-Boneh E., Froom P., Harari G., Malik M., Ribak J. (2000). Summer-winter differences in 24 h variability of heart rate. J. Cardiovasc. Risk.

[58] Markov A., Solonin I., Bojko E. (2016). Heart rate variability in workers of various professions in contrasting seasons of the year. Int. J. Occup. Med. Environ. Health.

[59] Pitzalis M., Mastropasqua F., Massari F., Forleo C., Di Maggio M., Passantino A., Colombo R., Di Biase M., Rizzon P. (1996). Short-andlong-term reproducibility of time and frequency domain heart rate variability measurements in normal subjects. Cardiovasc. Res..

[60] Driscoll D., DiCicco G. (2000). The effects of metronome breathing on the variability of autonomic activity measurements. JMPT.

[61] Reland S., Ville N., Wong S., Carrault G., Carre F. (2005). Reliability of heart rate variability in healthy older women at rest and during orthostatic testing. Aging Clin. Exp. Res..

[62] Bertsch K., Hagemann D., Naumann E., Schächinger H., Schulz A. (2012). Stability of heart rate variability indices reflecting parasympathetic activity. Psychophysiology.

[63] Zollei E., Csillik A., Rabi S., Gingi Z., Rudas L. (2007). Respiratory effects on the reproducibility of cardiovascular autonomic parameters. Clin. Physiol. Funct. Imaging.

